# Incidence and criteria used in the diagnosis of hospital-acquired malnutrition in adults: a systematic review and pooled incidence analysis

**DOI:** 10.1038/s41430-022-01141-2

**Published:** 2022-05-02

**Authors:** Liliana Botero, Adrienne M. Young, Merrilyn D. Banks, Judy Bauer

**Affiliations:** 1 School of Human Movement and Nutrition Science, The University of Queensland, Brisbane, QLD Australia; 2 Department of Nutrition and Dietetics, The Royal Brisbane and Women’s Hospital, Brisbane, QLD Australia; 3 Centre for Health Services Research, The University of Queensland, Brisbane, QLD Australia; 4 School of Nutrition, Dietetics & Food, Monash University, Melbourne, VIC Australia

**Keywords:** Nutrition, Malnutrition

## Abstract

Despite advances in identifying malnutrition at hospital admission, decline in nutritional status of well-nourished patients can be overlooked. The aim of this systematic review was to investigate the incidence of hospital-acquired malnutrition (HAM), diagnostic criteria and health-related outcomes. PubMed, CINAHL, Embase and Cochrane Library were searched up to July 2021. Studies were included if changes in nutritional status was assessed with a validated nutrition assessment tool in acute and subacute adult (≥18 yrs) hospitalised patients. A random-effects method was used to pool the incidence proportion of HAM in prospective studies. The certainty of evidence was appraised using the Grading of Recommendation Assessment, Development and Evaluation system. We identified 12 observational cohort studies (10 prospective and 2 retrospective), involving 35,324 participants from acute (9 studies) and subacute settings (3 studies). Retrospective studies reported a lower incidence of HAM (<1.4%) than prospective studies (acute: 9–38%; subacute: 0–7%). The pooled incidence of HAM in acute care was 25.9% (95% confidence interval (CI): 17.3–34.6). Diagnostic criteria varied, with use of different nutrition assessment tools and timeframes for assessment (retrospective studies: >14 days; prospective studies: ≥7 days). Nutritional decline is probably associated with longer length of stay and higher 6-month readmission (moderate certainty of evidence) and may be association with higher complications and infections (low certainty of evidence). The higher incidence of HAM in the acute setting, where nutritional assessments are conducted prospectively, highlights the need for consensus regarding diagnostic criteria and further studies to understand the impact of HAM.

## Introduction

The high prevalence of malnutrition in hospitalised patients is now well established [1–3]. Over the past two decades, great advances in the recognition and management of malnutrition have been made [4–6], which came with the development of several malnutrition assessment tools and criteria to aid in the recognition and diagnosis of malnutrition, such as the Subjective Global Assessment (SGA) [7], the Patient-Generated Subjective Global Assessment (PG-SGA) [8], the Mini Nutritional Assessment (MNA) [9], and the Global Leadership Initiative on Malnutrition (GLIM) criteria [5]. These tools and criteria include multiple domains to assess nutritional status, including weight loss, suboptimal intake, loss of muscle mass and/or subcutaneous fat as well as burden of disease [5, 7–9]. Best practice guidelines recommend that several distinct but interrelated steps need to be performed to effectively identify and treat malnutrition throughout hospital admission [5, 6, 10, 11]. These guidelines include routine malnutrition risk screening, nutrition assessment and tailored nutrition intervention to correct or prevent further nutritional decline [6, 10, 12]. Despite these advances, hospital malnutrition continues to be a highly prevalent problem [13–16] that may go be undetected, undiagnosed and undertreated [17–19].

The development of malnutrition in hospital inpatients is multifaceted. Disease and inflammation can be a major contributor to nutritional decline in hospital due to its effects on appetite, satiety, nutrient intake and absorption, and metabolic changes causing tissue breakdown [4–6, 20]. However, other factors such as dislike of hospital food [21], and operation-related processes such as being Nil By Mouth or restrictive diets [21, 22] can also contribute to inadequate intake of energy, protein and other nutrients [6]. Malnutrition has a significant impact on patients and the health care system. It contributes to morbidity, mortality, increased length of hospital stays and re-admission rates, decreased quality of life, physical function and higher health care costs [3–7]. In response to this, malnutrition was added to the list of hospital-acquired complications (HACs) by the Australian Commission in Quality and Safety in Health Care in 2018. They have defined HACs as a subset of healthcare related events which originate during a patient’s hospital stay and which were not present when the patient is admitted [23]. This definition, therefore, does not consider patients who experience further nutritional decline when they enter hospital already malnourished which would also suggest that nutrition care systems are lacking. In addition, this definition does not specify a diagnostic criteria or timeframe that should be considered to diagnose HAM.

While it is acknowledged that patients may experience nutritional decline during admission, to date, the incidence has not been well documented. Currently there is a lack of a consensus about what constitutes hospital-acquired malnutrition (HAM) and the criteria most appropriate to detect acute changes in nutritional status. Therefore, this systematic review sought to answer the following questions: How is HAM in adults defined?; What is the published incidence of HAM in adult hospitalised patients?; What validated criteria or nutrition assessment tool were used to diagnose HAM?; and, are there any health-related outcomes associated with HAM or in-hospital nutritional decline?

## Methods

**Fig. 1:**
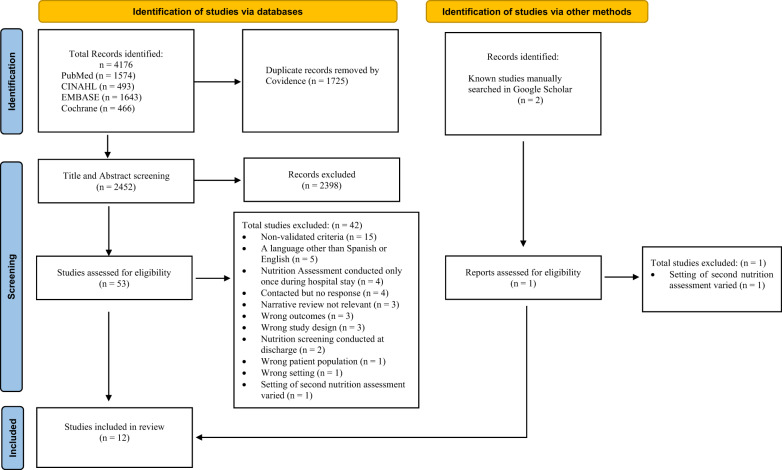
PRISMA 2020 flow diagram for new systematic reviews that included searches of databases and other sources. From: Page MJ, McKenzie JE, Bossuyt PM, Boutron I, Hoffmann TC, Mulrow CD, et al. The PRISMA 2020 statement: an updated guideline for reporting systematic reviews. BMJ 2021;372:n71. 10.1136/bmj.n71. For more information, visit: http://www.prisma-statement.org/.

We performed a systematic review of studies investigating nutritional decline or incidence of malnutrition during a hospital admission, including both acute and subacute (e.g. rehabilitation) admissions. The protocol was registered with PROSPERO International Prospective Register of Systematic Reviews (CRD42020206198), and it was reported in accordance to the Preferred Reporting Items for Systematic Reviews and Meta-Analysis (PRISMA) 2020 Statement [24], including the PRISMA 27-item checklist (Appendix a), the PRISMA 2020 flow diagram (Fig. 1).

### Search strategy

A systematic search of English and non-English journal articles was conducted in September 2020 using PubMed, CINAHL (via EBSCO*host*), EMBASE and the Cochrane Controlled Trials Register. The search strategy was developed, tested and refined in assistance with an academic librarian until the search result provided comprehensive and accurate articles. The search strategy was applied in PubMed as follows: *(“hospital-acquired malnutrition”[tiab] OR “hospital acquired malnutrition”[tiab] OR “hospital malnutrition”[tiab] OR “nosocomial malnutrition” [tiab]) OR (“nutritional status”[tiab] AND (“decline”[tiab] OR “change”[tiab] OR “changes”[tiab] OR “deterioration”[tiab]) AND (“Hospitalization”[Mesh] OR “hospitalization”[tiab] OR “hospitalisation”[tiab] OR “hospital”[tiab] OR “Hospitals”[Mesh] OR “hospitalised”[tiab] OR “hospitalized”[tiab] OR “nosocomial”[tiab] OR “Inpatients”[Mesh] OR “inpatients”[tiab] OR “in-patients”[tiab]))*, with the same key terms applied for all the other databases (Appendix b). The search was limited to exclude “*child”* or “*pediatrics”* and animal studies. Due to the large number of results, CINAHL was further limited by year (1990–2020), and narrowed by adult only, and EMBASE was limited to journal articles only. The reference lists of included papers were reviewed; however, no new papers were identified from this method. In addition, the search was repeated in July 2021 in all databases to identify if any new studies had been published since the original search. No new articles were identified in the last search.

### Eligibility criteria: participants, setting, nutrition assessment and types of studies

Studies were included if they assessed changes in nutrition status in adult patients (aged ≥ 18 yrs) admitted to an acute or subacute hospital ward and the nutrition assessment was conducted at two time points during hospitalisation with a validated nutrition assessment tool (e.g. Subjective Global Assessment [7] or other tool, or criteria as recommended by the Global Leadership Initiative on Malnutrition (GLIM) [25] or the International Classification of Disease Australian Modification, tenth revision (ICD-10-AM) [26]). Studies were excluded if they used only individual anthropometric nutrition parameters (such as weight only, muscle mass or fat mass only, or hand-grip strength only) or biochemical markers (such as albumin, lymphocyte count) to assess nutritional status [6]. Body Mass Index (BMI) was included as a criterion for nutrition assessment, consistent with ICD-10-AM, only if BMI was categorised as underweight (BMI < 18.5 kg/m^2^) vs normal weight or overweight (BMI ≥ 18.5 kg/m^2^) to allow evaluation of changes in nutrition status. Narrative reviews, conference abstracts, thesis articles, paediatrics, language other than English or Spanish, or studies conducted in non-inpatient settings (e.g. nursing homes, outpatients, emergency department) were also excluded. Only peer-reviewed journal articles were selected in order to provide the strongest evidence to help answer the question for this systematic review [27].

### Data management and screening process

All identified articles were imported into the online platform Covidence Systematic Review Software (www.covidence.org) for automated removal of duplicates, and screening and selection by the authors. Screening of studies was conducted in two phases. In Step 1, one author (LB) scanned all titles and abstracts of identified studies for potential eligibility. In the case where there was insufficient detail in the title or abstract, the article was moved into full text review for further examination. In Step 2, the full text of all potentially eligible studies were reviewed by two independent authors (LB and AY), and those that met the pre-defined selection criteria were included for data extraction. During the full text screening, conflicts for one article [28] were resolved by a third author (MB).

Due to the lack of consensus on the definition of adult “hospital-acquired malnutrition” [21, 29], all studies that evaluated decline or change in nutritional status during hospitalisation and provided sufficient data to extract the incidence of HAM were included in this review.

### Data extraction

Data was manually extracted by one author (LB) and reviewed independently by a second reviewer (AY). Where additional information was required, the relevant author/s were contacted with a follow up email four weeks from the initial contact. Studies were excluded if a response was not provided after two weeks from the second email (i.e. more than 6 weeks from initial contact). The following information was extracted from each study: author(s), article title and year, aim of the study, location, setting (acute/subacute), participant characteristics (including: sample size, mean and standard deviation (SD) or median age and interquartile ranges (IQR) for age and length of stay (LOS); number and percentage of males and females; inclusion and exclusion criteria), nutrition assessment tool or criterion used to assess changes or decline in nutritional status, nutrition assessment time points, incidence of HAM, prevalence of malnutrition on admission and at discharge, rate of decline and improvement of nutritional status, health outcomes if reported, definition of HAM if provided, funding source, and conclusion of the study. All data was extracted onto a Microsoft^®^ Excel spreadsheet.

### Extraction of the incidence of hospital-acquired malnutrition

The Australian Commission on Quality and Safety in Healthcare’s definition for HAM was applied in this study. The incidence of HAM was either reported from the study, extracted, or re-calculated (if malnourished patients at baseline had been included in the denominator in the individual studies) using the below formula:

$${{{\mathbf{HAM}}}}\;{{{\mathbf{Incidence}}}}\;{{{\mathbf{Proportion}}}} = \left( {\frac{{{{{\mathrm{number}}}}\;{{{\mathrm{of}}}}\;{{{\mathrm{patients}}}}\;{{{\mathrm{who}}}}\;{{{\mathrm{developed}}}}\;{{{\mathrm{HAM}}}}\;{{{\mathrm{at}}}}\;{{{\mathrm{follow}}}}\;{{{\mathrm{up}}}}\;{{{\mathrm{assessment}}}}}}{{{{{\mathrm{number}}}}\;{{{\mathrm{of}}}}\;{{{\mathrm{at}}}}\;{{{\mathrm{risk}}}}\;{{{\mathrm{or}}}}\;{{{\mathrm{well}}}} - {{{\mathrm{nourished}}}}\;{{{\mathrm{patients}}}}\;{{{\mathrm{at}}}}\;{{{\mathrm{baseline}}}}}}} \right) \times 100$$

### Quality appraisal and certainty of the evidence

Quality of the methodology and risk of bias were assessed using the Academy of Nutrition and Dietetics’ (AND) Quality Criteria Checklist for Primary Research tool [27]. Each study received a positive, neutral or negative rating depending on the extent to which the study had addressed the quality criteria. The quality assessment was independently conducted by two authors (LB and AY) and discrepancies were discussed between three authors (LB, AY and JB). The overall certainty of the evidence for each listed outcomes was assessed using the Grading of Recommendation Assessment, Development and Evaluation (GRADE) Criteria [30], which considers the study design, the risk of bias, imprecision, inconsistency and indirectness. This was conducted by two authors (LB and JB).

### Pooled incidence data analysis and synthesis

The pooled incidence proportion of HAM was undertaken in Cochrane Review Manager (RevMan) version 5.4.1 using the DerSimonian and Laird random effects model with inverse-variance weights without alterations [31]. Heterogeneity amongst the included studies was calculated by means of the inconsistency index I^2^, where a I^2^ value of >50% indicates a high heterogeneity amongst the included studies [32]. Two retrospective studies [21, 29] were not included in the pooled analysis due to the difference in study designs between prospective and retrospective studies.

## Results

### Search results and included studies

Figure 1 summarises the search results and included studies. A total 4176 studies were identified from electronic databases and two related studies known to the authors were manually added. After removing 1725 duplicates, there were 2452 studies identified for title and abstract screening. A total of 54 studies were included in the full-text review; of these, eight authors were contacted for additional information [21, 29, 33–38]. Of the authors who were contacted, two [21, 29] were contacted to seek clarification in the methodology used to identify the incidence of HAM, and six [33–38] were contacted for additional data to extract the incidence of HAM. Four studies [35–38] were excluded from this review as additional data were not provided by the authors.

Twelve studies met the inclusion criteria (Fig. 1), published between 2000–2021 and conducted in Australia (*n* = 7), Europe (*n* = 2), North America (*n* = 2) and South America (*n* = 1).

### Study design and study quality

Table 1 shows the characteristics of the included studies, of which eight were undertaken in the acute care setting [21, 28, 33, 39–43], three in subacute [34, 44, 45], and one across both acute and subacute combined [29]. All 12 studies were observational; of these, two were retrospective clinical audits [21, 29] and ten were prospective cohort studies [21, 28, 33, 34, 39–45]. Using the Quality Criteria Checklist for Primary Research, seven of the ten studies were rated positive [33, 34, 39, 40, 42, 44, 45], two neutral [28, 41], and three negative [21, 29, 43] (Table 2).

**Table 1:** Study characteristics from studies evaluating in-hospital changes and decline in nutritional status as assessed with a validated nutrition assessment tool.

Author, year. Location	Study design	Participant characteristics	Validated nutrition assessment tool/criteria	Other individual nutritional markers used in the study	Timeframe between nutrition assessments	Reported/extracted^d^ incidence of HAM in percentage (*n*)	Total rate of nutrition status decline^e^ and improvement^e^ in percentage (*n*)	Malnutrition prevalence in percentage (*n*)	Outcomes associated with nutritional decline
*Acute*									
Lima J, et al. 2021 [42] Brazil	Secondary analysis of a prospective cohort study	*N* = 299 48% males Mean age: 57 yrs (±14.6) Mixed acute patient group, excluding: palliative, ICU, cognitive impairment, oedema, bed-ridden, unable to answer questions, or a LOS of <3 days.	SGA	Severe weight loss of ≥2% within the first week	At admission (within 48 h) and 7 days after admission (within the first week)	9% (16/171)^d^	Decline: 28% (48/171) Improvement: 3% (4/128)	Admission: 43% (128/299) Discharge: 53% (159/299)	Increased risk of prolonged LOS (*p* = 0.034), and re-admission (*p* = 0.041) for patients who declined in SGA category within the first week (after adjusting for CCI and SGA category on admission)
van Vliet I, et al. 2020 [43] The Netherlands	Prospective observational study	*n* =91/584 46% males Mean age: 61 yrs (± 15.1) Surgical, medical wards, excluding: palliative, delirium, LOS <1 day, patients in isolation or not able to understand Dutch/English	PG-SGA	N/A	At admission (within 24 h) and at discharge, median LOS was 8 days (IQR 6–9)	30% (17/57)	Decline: 40% (23/57) Improvement: 29% (10/34)	Admission: 37% (34/91) Discharge: 49% (45/91) (from longitudinal assessments only)	Not investigated
Cheng J, et al. 2019 [21] Australia	Retrospective audit	*N* = 15,419 78% males Median age: 59 (19–83) All acute admissions	SGA, if SGA was not completed, the authors used components of the SGA	N/A	Not reported	0.15% (23/15,023)^d^	Not reported	2.7% (416/15,419) - Time not specified	Not investigated
Allardb J. P. et al. 2016 [39] Canada	Prospective multi-centre observational study	*N* = 409 53% males Median age: 68 yrs (58–79) Surgical, medical wards, excluding: palliative, ICU, obstetric, psychiatric, or medical day unit; LOS <7 days	SGA	Weight loss of ≥5%	At admission (within 48 h) and discharge, median LOS 11 days (IQR 8–17)	31% (61/200)^d^	Decline: 41% (81/200) Improvement: 34% (71/209)	Admission: 51% (209/409) Discharge: 54% (220/409)	Longer LOS for patients who declined from SGA A (*p* = 0.005) and from SGA B (*p* < 0.001) after adjusting for confounding variables^g^
Hung Y.C, et al. 2013 [28] Australia	Pilot observational study	*N* = 24 71% males Mean age: 56 (±13) Oncology: high-dose conditioning and autologous peripheral blood stem cell transplantation (PBSCT), excluding allogenic PBSCT	PG-SGA	Lean body mass, fat mass, hand-grip strength.	At pre-admission (5 ± 4 days before admission), and at hospital discharge, mean LOS 23 ± 7 days	30% (7/23)^d^	Decline**:** 30% (7/23) Improvement**:** 0% (0/23)	Admission: 4% (1/24) Discharge: 35% (8/23)	Not investigated
Bell, J, et al. 2012 [40] Australia	Prospective observational study	*N* = 44 27% Males Mean age: 82 yrs (±11) Hip fracture patients admitted for surgical intervention, nil exclusions	ASPEN/the Academy 2012 Criteria	N/A	At admission (within 72 h), and at discharge, Median (range) LOS was 14 days (32 days)	24% (5/21)^d^	Decline: 39% (9/23) Improvement: 0% (0/21)	Admission: 52% (23/44) Discharge: 64% (28/44)	Not investigated
Cansado P, et al. 2009 [33] Portugal	Prospective observational study	*N* = 531 56% males Median age: 73 yrs (65–91) Geriatric medical and surgical wards, excluding: palliative, bed-ridden, unable to answer questions, or a LOS of <3 days	MNA, and a LOS of >3 days	BMI, MUST (for screening)	At admission and discharge, mean LOS: 12 days (± 10) (range: 3–62)	24% (84/359)^c,d^	Decline: 24% (84/359)^f^ Improvement: 10% (44/440)^f^	Admission: 32% (172/531) Discharge: 40% (212/531)	Not investigated
Braunschweig C, et al. 2000 [41] USA	Prospective observational study	*N* = 404 49% males Mean age: 54 yrs (0.82) Acute wards not specified, excluding: pregnant or lactating women, or psychiatric patients	SGA	N/A	At admission (within 72 h) and discharge, median LOS 12 days, mean 17 (± 0.61)	38% (70/185)	Decline: 68% (126/185) Improvement: 39% (86/219)	Admission: 54% (219/404) Discharge: 59% (238/404)	Increased odds of complications was significantly higher (*p* ≤0.05) for patients who declined from SGA A to B or C, from SGA B to C, and from SGA C with >5% weight loss. Both LOS^h^ and infections^i^ were only significantly associated with nutritional decline in univariate analysis.
Woodward T, et al. 2020 [29] Australia	Retrospective audit	*N* = 17,717 55% males Mean age: 63 yrs (± 20) All acute and subacute^a^ admissions	SGA (*n* = 104/208 patients received an initial SGA [46]^b^, therefore clinical judgement using components of SGA was applied to diagnose HAM).	N/A	Patients admitted for > 14 days who appeared, or were assessed as well-nourished on admission	1.4% (208/14,701)^d^	Not reported	Admission: 17% (3016/17,717) Discharge: not reported	Only descriptors or patient characteristics associated with the development of HAM were reported.
*Subacute*^a^									
Whitley A., et al. 2017 [45] Australia	Prospective observational study	*N* = 59 46% males Mean: 84 yrs (±6.6) Geriatric subacute^a^ ward, excluding: palliative care, length of stay ≤21 days, those who declined a nutrition assessment within 72 h of admission	SGA	N/A	At admission^c^ (within 72 h) discharge (72 h prior), median LOS 34 days (IQR: 26–43)	7% (2/27)^d^	Decline: 7% (2/27) Improvement: 40% (13/32)	Admission: 54% (32/59) Discharge: 44% (26/59)	Not investigated^j^
Collins, J, et al. 2016 [34] Australia	Prospective observational study	*N* = 213 37% males Median: 80 yrs (73–84) Geriatric subacute^a^ ward, exclusion: none reported due to waiver of consent received for this study	MNA	Weight, mid arm and calf circumference, hand-grip strength and fat free mass.	At admission^c^ (within 72 h) and discharge (72 h prior), median LOS 17 (IQR 12–29)	7% (11/151)^d^	Decline: 13% (22/175) Improvement: 29% (59/204)	Admission: 29% (62/213) Discharge: 20% (42/213)	Not investigated
McDougall et al. 2015 [44] Australia	Prospective observational study	*N* = 14 44% males Mean: 83 yrs (± 7) Geriatric subacute^a^ ward, excluding: palliative and a LOS of <14 days.	MNA	N/A	At admission^c^ (within 72 h) and as close as possible to day 21, mean day 21 (±2.4)	0% (0/11)	Decline: 4% (3/77) Improvement: 29% (30/103)	Admission: 32% (37/114) Discharge: 19% (22/114)	Not investigated

*HAM* hospital-acquired malnutrition, *ICU* intensive care unit, *IQR* interquartile range, *SGA* Subjective Global Assessment, *PG-SGA* Patient Generated Subjective Global Assessment, *MNA* the Mini Nutritional Assessment, *ASPEN* American Society for Parenteral and Enteral Nutrition, The Academy the Academy of Nutrition and Dietetics, *MUST* Malnutrition Universal Screening Tool, *LOS* length of stay, *NS* nutrition status, *N/A* not applicable, *CCI* Charlson’s Comorbidity Index.

^a^Subacute refers to multidisciplinary care provided to post-acute patients to optimise function and quality of life [58].

^b^Additional information regarding number of patients assessed with the SGA was obtained from a recently published paper by the same authors using the same data [46].

^c^Subacute admission.

^d^Incidence of HAM if not obtained directly from the article, it was extracted / recalculated based on data reported in the study or as provided by the author/s. This was calculated with the formula: [(number of new cases/number of well-nourished on admission or population at risk who were not been malnourished) × 100].

^e^A decline in nutritional status, as assessed with the MNA can be a decline from well-nourished to both malnutrition risk and malnourished state, and from malnutrition risk to being malnourished, and an improvement in nutritional status can be an improvement from malnourished to being at risk or to being well-nourished. As assessed with the SGA or PG-SGA, a decline in nutritional status can be a decline from SGA/PG-SGA A to B or C, or from SGA/PG-SGA B to C, and an improvement can be from SGA / PG-SGA C to B or A, or from SGA/PG-SGA B to A.

^f^HAM, nutrition decline and nutrition improvement were extracted from raw data provided by the author.

^g^Confounding variables included: age, gender, living arrangement, number of diagnostic criteria, Charlson Comobidity Index (CCI) at admission and change in CCI.

^h^No multivariate analysis was conducted for LOS.

^i^After multivariate analysis, increased odds of infections was not statistically significant with nutritional decline when compared to the reference group: stable well-nourished.

^J^Outcomes not included due to patients with nutritional decline and patients who remained stable being grouped together during statistical analysis.

**Table 2:** The Academy of Nutrition and Dietetics Quality Criteria Checklist (primary research) for studies included in this systematic review.

Reference	Overall Quality Rating^q^	Relevance Question^r^	Validity Questions^s^										Comments
			1	2	3^a^	4^a^	5^a^	6^a^	7	8	9	10	
Lima J, et al. 2021 [42]	(+) POSITIVE	Y	Y	Y	N/A	N/A	N/A	N/A	Y^b^	Y	Y	Y	• ^b^Repeated nutrition assessments done at day 7, this might not see sufficient time to observe changes in nutritional status when using the SGA.
van Vliet, et al. 2020 [43]	(−) NEGATIVE	Y	Y	U	N/A	N/A	N/A	N/A	N^c^	Y	Y	U^d^	• ^c^A practice-based study, unclear of inter-rater reliability • ^d^ Conflict of interest (if any) was not reported
Woodward T, et al. 2020	(−) NEGATIVE	Y	Y	N^e^	N/A	N/A	N/A	N/A	N^f^	Y	Y	U^g^	• ^e^Did not exclude palliative patients. • ^f^Unclear timeframe between nutrition assessments. Further, in the absence of the SGA, clinical judgement was used to assess decline in nutritional status; however, it was not reported what variables were assessed under clinical judgement. • ^g^Conflict of interest (if any) was not reported
Cheng J, et al. 2019 [21]	(−) NEGATIVE	Y	Y	N^h^	N/A	N/A	N/A	N/A	N^i^	Y	Y	Y	• ^h^Did not exclude palliative patients. • ^i^Unclear timeframe between nutrition Assessments. Further, in the absence of the SGA, components of the SGA were used; however, it was not reported which variables within the SGA were assessed.
Whitley A, et al. 2017 [45]	(+) POSITIVE	Y	Y	Y	N/A	N/A	N/A	N/A	Y	Y	Y	Y	
Collins J, et al. 2016 [34]	(+) POSITIVE	Y	Y	Y	N/A	N/A	N/A	N/A	Y	Y	Y	Y	
Allard J. P, et al. 2016 [39]	(+) POSITIVE	Y	Y	Y	N/A	N/A	N/A	N/A	Y^j^	Y	Y	Y	• ^j^Median LOS was 11 days, which might not see sufficient time to observe changes in nutritional status when using the SGA.
McDougall et al. 2015 [44]	(+) POSITIVE	Y	Y	Y	N/A	N/A	N/A	N/A	Y	Y	Y	Y	
Yun-Chi Hung et al, 2013	Ø NEUTRAL	Y	Y	Y	N/A	N/A	N/A	N/A	N^k^	Y	Y	Y	• ^k^baseline nutrition assessment in some patients was conducted 23 or 33 days before hospitalisation, therefore unable to determine if nutritional decline did not occur prior to hospital admission.
Bell et al. 2012	(+) POSITIVE	Y	Y	Y	N/A	N/A	N/A	N/A	Y	Y	Y	Y	• Conflict of interest (if any) was not reported
Cansado P, et al. 2000	(+) POSITIVE	Y	Y	Y	N/A	N/A	N/A	N/A	Y^l^	Y	U^m^	Y	• ^l^The median LOS was 11 days, and the minimum timeframe between assessments was 3 days, which might not see sufficient to observe changes in nutritional status when using the SGA. • ^m^Biases and limitations were not identified and discussed in the study
Braunschweig C, et al. 2000 [41]	Ø NEUTRAL	Y	Y	Y^m^	N/A	N/A	N/A	N/A	Y^n^	Y^o^	Y	U^p^	• ^n^Complications and infections were not defined in the study • ^o^Adjustments for confounding factors were not made for all outcomes (specifically, length of stay). Mean (SD) and Median (without interquartile ranges) were presented for the same continuous variables. • ^p^Sources of funding and conflict of interest (if any) was not reported • ^m^Did not exclude palliative patients

*Y* yes, *N* no, *U* unclear, *N/A* not applicable, *SGA* Subjective Global Assessment, *MNA* the Mini Nutritional Assessment, *LOS* length of stay, *SD* standard deviation.

^a^Due to the nature of the studies included, validity questions 3,4,5, and 6 were not applicable.

^q^Assignment of Overall Quality Rating:

MINUS/NEGATIVE (−) If most (six or more) of the answers to the above validity questions are “No,” the report should be designated with a minus (−) symbol on the Evidence Worksheet

NEUTRAL (∅) If the answers to validity criteria questions 2, 3, 6, and 7 do not indicate that the study is exceptionally strong, the report should be designated with a neutral (∅) symbol on the Evidence Worksheet.

PLUS/POSITIVE ( + ) If most of the answers to the above validity questions are “Yes” (including criteria 2, 3, 6, 7 and at least one additional “Yes”), the report should be designated with a plus symbol (+) on the Evidence Worksheet.

^r^Relevance Questions (*n* = 4).

1. Would implementing the studied intervention or procedure (if found successful) result in improved outcomes for the patients/clients/population group? (NA for some Epi studies)

2. Did the authors study an outcome (dependent variable) or topic that the patients/clients/population group would care about?

3. Is the focus of the intervention or procedure (independent variable) or topic of study a common issue of concern to dietetics practice?

4. Is the intervention or procedure feasible? (NA for some epidemiological studies)

^s^Validity Questions (*n* = 10).

1. Was the research question clearly stated?

2. Was the selection of study subjects/patients free from bias?

3. Were study groups comparable?

4. Was method of handling withdrawals described?

5. Was blinding used to prevent introduction of bias?

6. Were intervention/therapeutic regimens/exposure factor or procedure and any comparison(s) described in detail?; Were intervening factors described?

7. Were outcomes clearly defined and the measurements valid and reliable?

8. Was the statistical analysis appropriate for the study design and type of outcome indicators?

9. Are conclusions supported by results with biases and limitations taken into consideration?

10. Is bias due to study’s funding or sponsorship unlikely?

### Participant characteristics

Participant characteristics are displayed in Table 1. In total there were 35,324 adult hospitalised patients across the 12 studies. In the prospective studies (in the acute and subacute setting), the sample size was between 24 to 584 adult inpatients [28, 33, 34, 39–45], while the two retrospective studies [21, 29] included 15,419 and 17,717 hospital admissions respectively. Of the acute prospective studies, three recruited patients from medical and surgical wards [33, 39, 43], one recruited oncology patients undergoing high-dose conditioning and autologous peripheral blood stem cell transplantation [28], and one recruited acute hip fracture patients admitted for surgical intervention [40]. The remaining two acute prospective studies, failed to specify the acute ward or unit where participants were recruited [41, 42]. All three prospective subacute care studies included patients aged ≥65 yrs admitted to geriatric evaluation and management units in Australia [34, 44, 45].

### Definition of hospital-acquired malnutrition

Of the twelve included studies, only two provided specific definitions of HAM [21, 29]. The remaining ten studies evaluated changes in nutritional status, hence, a definition of HAM was not provided. Cheng and colleagues (2019) [21] defined HAM as “any decline in nutritional status that occurs during hospital stay, independently of nutritional status on admission, which was further categorised as “preventable” and “non-preventable” [21]. “Preventable HAM” was defined as a “decline in nutritional status in the absence of injury or inflammation (starvation related malnutrition), or a decline in nutritional status in the presence of injury or inflammation but received inadequate nutrition for the condition (disease-related malnutrition)”. While “non-preventable HAM” was defined as a “decline in nutritional status in the presence of injury or inflammation and received adequate nutrition for the condition (disease-related malnutrition)” [21]. Woodward and colleagues on the other hand, defined HAM as “malnutrition first diagnosed >14 days after admission” [29].

### Incidence of hospital-acquired malnutrition and criteria used in its diagnosis

Overall, acute prospective studies reported an incidence of HAM between 9%–38% [28, 33, 39–43]. This is higher than that reported in retrospective studies in the acute setting (<1.4%) [21, 29] and in prospective subacute studies, which either found no cases of HAM [44] or found a HAM incidence of 7% [34, 45] (Table 1). In the acute prospective studies, it was observed that the longer the timeframe between nutrition assessment, the higher the incidence of HAM [28, 33, 39, 41, 43].

**Fig. 2:**
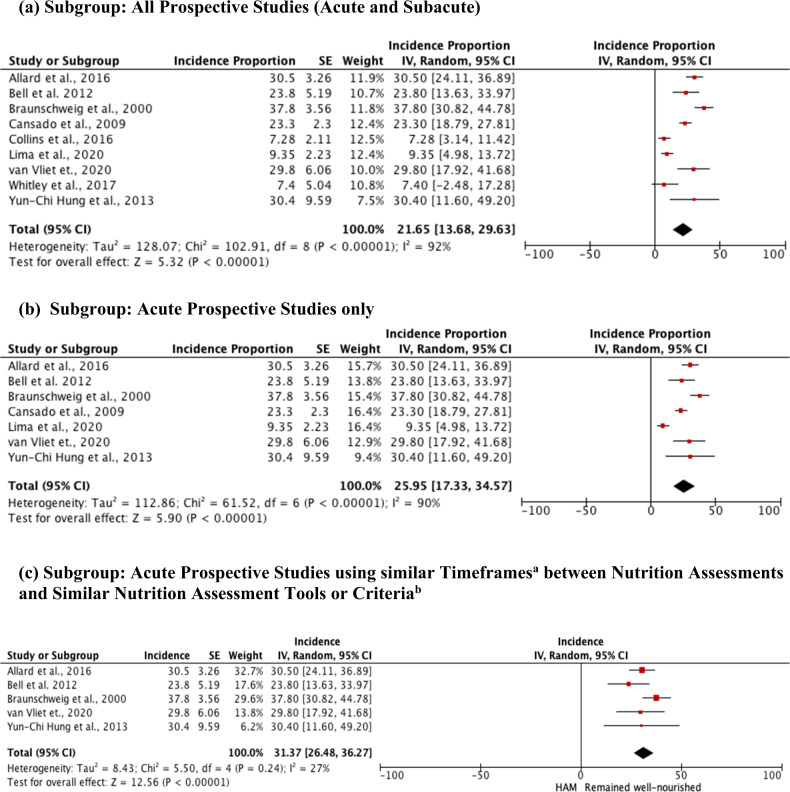
Forest plots for cumulative incidence proportion of HAM for prospective studies. ^a^Baseline assessment conducted 5 (±4 days) prior admission or up to 72 h of admission, and follow up assessment conducted at discharge. ^b^Nutrition assessment tools or criteria included are: the Subjective Global Assessment; the Patient Generated Subjective Global Assessment; and the 2012 malnutrition diagnostic criteria by the Academy of Nutrition and Dietetics and the American Association of Enteral and Parenteral Nutrition. CI confidence interval, SE standard error, IV inverse variance, df degrees of freedom.

The calculated pooled incidence proportion of HAM among all the prospective studies was 21.65% (95% Confidence Interval (CI) 13.68, 29.63) with a high heterogeneity amongst the studies (*I*^2^ = 92%) (Fig. 2a). When only prospective studies conducted in the acute setting were included, the pooled incidence proportion of HAM was 25.95% (95% CI = 17.33, 34.57), however, heterogeneity remained high (*I*^2^ = 90%) (Fig. 2b). Further subgroup analyses were conducted to explore causes of heterogeneity. When only acute prospective studies, which used a similar timeframe between assessments and similar nutrition assessment tools or criteria were included, the incidence proportion of HAM was 31.37% (95% CI = 26.48–36.27) with a low heterogeneity (*I*^2^ = 27%) (Fig. 2c).

Of the ten prospective studies included in this review, the SGA was used in six studies (as a stand-alone assessment or within the PG-SGA) [28, 39, 41–43, 45], the MNA was used by three studies [33, 34, 44], and one study used the 2012 malnutrition diagnostic criteria by the Academy of Nutrition and Dietetics and the American Association of Enteral and Parenteral Nutrition [40]. The average timeframe between nutrition assessment in acute prospective studies ranged between 7–23 days, and in the subacute studies between 17–34 days [34, 44, 45] (Table 1).

The two retrospective studies reported using the SGA to identify cases of HAM [21, 29]; however, they did not specify the number of patients who were assessed with this tool (as opposed to having the tool applied retrospectively based on information available in the medical record), and, for those who were assessed with the SGA, it was not clarified whether repeated nutrition assessments had been conducted and when. Additional information was obtained for the Woodward et al. (2020) study [29] from a recently published article which used the same data [46]. This study reported that out of the 208 patients who were clinically assessed to have developed HAM, only half (*n* = 104/208) were initially assessed with the SGA, however, no follow up assessment with the SGA was reported to have been performed [46].

### Health outcomes associated with in-hospital nutritional decline

Three acute prospective studies found that increased length of stay (LOS) was significantly associated with nutrition status decline (independently of nutrition status on admission) [39, 41, 42]. After adjusting for confounding variables, two studies showed that LOS was still significantly longer for patients with decline in nutritional status [39, 42]. One [41] did not conduct a multivariable statistical analysis for this specific outcome, making it difficult to establish if decline in nutritional status was an independent risk factor for increased LOS.

From the Braunschweig et al (2000) study [41], patients who either experienced nutritional decline, remained malnourished, or were admitted to hospital malnourished but improved from SGA C to B and from B to A, incurred higher costs (of almost $US 10,000–$38,000 more) than patients who remained well-nourished; however univariate analysis found this to be statistically significant only for those patients who declined from SGA A to C (*p* ≤ 0.05) [41]. A multivariate analysis showed higher odds of complications for patients who experienced a decline from SGA A or B to C [odds ratio (OR) = 3.8 (CI 1.2,11.4) and OR = 2.4 (CI 1.0, 5.9) respectively], and from SGA C with a further 5% weight loss (OR = 3.1 (CI 1.3, 7.4) [41]. However, the authors failed to describe the types of complications identified in the study.

Lima and colleagues [42] evaluated if in-hospital mortality, 6-month mortality and 6-month hospital readmission was associated with decline in SGA category in the first week of acute admission. Only 6-month readmission rates were significantly higher for patients with decline in nutritional status (*p* = 0.03); this was still significantly higher after a multivariate analysis [OR = 3.59 (CI 1.05, 12.26)] [42].

### Certainty of the evidence

Table 3 summarises the GRADE certainty of the evidence for the association between in-hospital nutritional decline and each outcome identified in the acute prospective studies [39, 41, 42]. The certainty of the evidence was moderate for LOS [39, 41, 42] and re-admission in 6 months [42], and low for infections and complications [41]. The certainty of evidence was downgraded due to serious bias (neutral quality of one study [41]) and imprecision due to the limited sample sizes/events.

**Table 3:** Certainty of Evidence for Outcomes Associated with In-Hospital Nutritional Decline.

	Certainty Assessment^a^						
Outcome	Number of Participants (Studies)	Risk of bias	Inconsistency	Indirectness	Imprecision	Publication bias	GRADE^e^
Length of stay	1112 (3 observational prospective studies) [39, 41, 42]	Serious^b^	Not serious	Not serious	Not serious	Undetected	Moderate ⨁⨁⨁◯
Readmission in 6 months	299 (1 observational prospective study) [42]	Not serious	Not serious	Not serious	Serious^c^	Undetected	Moderate ⨁⨁⨁◯
Complications	404 (1 observational prospective study) [41]	Serious^d^	Not serious	Not serious	Serious^c^	Undetected	Low ⨁⨁◯◯
Infections	404 (1 observational prospective study) [41]	Serious^d^	Not serious	Not serious	Serious^c^	Undetected	Low ⨁⨁◯◯

^a^Certainty of the evidence assessed using the Grading of Recommendation Assessment, Development and Evaluation (GRADE) criteria.

^b^evidence downgraded due the quality of the evidence being neutral for one study (Table 3).

^c^evidence downgraded due to low number of observations/events.

^d^evidence downgraded due the quality of the evidence being neutral for this study (Table 3).

^e^GRADE Working Group grades of evidence:

⊕⊕⊕⊕High Quality: Further research is very unlikely to change our confidence in the estimate of the effect.

⊕⊕⊕○Moderate Quality: There is moderately confidence in the effect estimate: the true effect is likely to be close the estimate of the effect, but there is a possibility that is substantially different

⊕⊕○○Low Quality: The confidence in the effect estimate is limited: the true effect may be substantially different from the estimate of the effect.

⊕○○○Very Low Quality (D): We have very little confidence in the effect estimate.

## Discussion

This systematic review aimed to determine the incidence and the criteria used in the diagnosis of HAM in the acute and subacute care. Despite the large volume of research being conducted in hospital malnutrition, only ten prospective studies were identified to have used a validated nutrition assessment tool or criteria at two timepoints to determine changes in nutritional status in acute and subacute patients. The incidence proportion of HAM amongst all included studies ranged between 0% and 38%. However, study design and setting appeared to influence the incidence of HAM, with subgroup pooled analysis including only acute prospective studies using a similar methodology finding an incidence of 31.37%, much higher than <1.4% incidence reported in the two retrospective audits. From the acute prospective studies, it was observed that the longer the timeframe between nutrition assessment, the higher the incidence of HAM. For example, Lima and colleagues [42], who assessed changes in nutritional assessment within the first week of admission with the SGA found a lower incidence of HAM compared to the other acute prospective studies using the same tool. This could be due to the fact that one week period between nutrition assessments is unlikely to observe deterioration in nutritional status.

The large contrast in the incidence of HAM between prospective and retrospective studies contributes to the body of evidence reporting that hospital malnutrition is highly undetected, undocumented and possibly undertreated [17–19]. In-hospital decline in nutritional status, if under-recognised, can lead to detrimental effects to patients’ outcomes due to malnutrition possibly being undertreated [3–7]. This may also impact on health services monitoring hospital-acquired complications, as the true incidence of HAM, and the magnitude of its associated effects (such as increased healthcare costs) are possibly largely underestimated. For example, the Australian Commission on Quality and Safety in Health Care, have reported the incidence of HAM as 0.12% [11], a much lower figure than the pooled incidence from prospective cohort studies in this review. As demonstrated by Braunschweig and colleagues [41], all groups of patients who experienced malnutrition (either on admission or acquired during hospital stay) incurred higher hospital charges (US $10,000–$38,000 more) than patients who remained well-nourished [41].

The studies conducted in subacute care also found a lower incidence of HAM (< 7%) compared to the acute prospective studies. The lower overall nutritional decline reported in the subacute studies could be attributed to likely lower acuity of the patients, experiencing less inflammation and possibly fewer nutrition impact symptoms. Patients included in these studies received active dietetic input during subacute stay; hence, potentially having better access to nutrition expertise and better nutrition care. Despite the lower incidence of HAM in this patient subgroup, the prevalence of malnutrition at discharge remained high (between 19% and 44%) [44, 45, 47], which has been shown to negatively impact patients function and quality of life once discharged to the community [48]. Therefore, there is a continual need to pay attention to nutritional status throughout the subacute admission, despite the lower incidence of HAM in this setting.

Hospital malnutrition has been associated with poorer outcomes. Malnourished patients experience longer LOS, increased readmission and mortality rates, higher risk of pressure ulcer [19, 20, 49–52]. Consistent with these findings, three studies included in this systematic review reported worse health outcomes for patients who experienced a decline in nutritional status compared to patients who remained well-nourished or improved nutritional status. Nutritional decline during acute stay was significantly associated with a longer LOS [39, 41, 42], increased odds of hospital readmission in 6 months [42], and increased risk of complications and infections [41]. Based on the GRADE certainty of the evidence, HAM is probably associated with longer LOS (moderate certainty: downgraded due to risk of bias) and 6-month readmission (moderate certainty: downgraded due to imprecision) and probably associated with higher infections and complications (low certainty: downgraded due to risk of bias and imprecision). Further prospective research is needed to increase the certainty of evidence regarding HAM and health outcomes including quality of life.

### Defining HAM

This systematic review found that the term HAM was only used in the two most recently published Australian papers, likely due to recent pricing and funding models applied to HAM since 2018 [53, 54]. The GLIM group was formed with the intention to seek a consensus on the diagnosis of malnutrition, nevertheless, GLIM criteria does not specify how to assess acute changes in nutritional status. Indeed, we found that studies included in this systematic review used different methods to evaluate changes in nutrition status during acute and subacute hospital stay, which resulted in a wide range in the incidence of HAM between prospective studies. This highlights the importance in gaining consensus on how to best measure acute changes or decline in nutritional status, as well as, to clarify the definition of HAM to create consistency in clinical research, for coding, and for costing and funding purposes [21].

In 2009, Jensen and colleagues proposed a classification of malnutrition based on aetiological factors: starvation-related malnutrition, disease-related malnutrition with inflammation (acute or chronic i.e. cachexia), and disease-related malnutrition without inflammation [55]. This aetiology-based classification has been endorsed by the 2012 AND / ASPEN guidelines [6], the ESPEN consensus statement [56], and by GLIM [5]. Because inadequate intake and inflammation play a major role in the development or worsening of malnutrition in hospitalised patients, further clarification is also needed on whether HAM should be included within this aetiology classification system.

### Limitations

While this was a comprehensive review of the literature, only studies that were published in English or Spanish were eligible for inclusion, limiting inclusion of studies published in other languages. In addition, four studies were excluded from this review due to lack of information to allow calculation of the incidence of HAM.

## Conclusions

The overall incidence of HAM ranged between 0.15–38%, which is much higher in acute care settings where prospective assessment of nutritional status were conducted (pooled incidence 25%). The low incidence of HAM in retrospective studies suggests that hospital malnutrition (present on admission and acquired) continues to be undetected, despite advances in the recognition and diagnosis of malnutrition. HAM is probably associated with increased LOS and 6-month readmission (moderate certainty of evidence) and may be associated with higher complications and infections (low certainty of evidence). The definition of HAM (criteria and timeframes between assessments) requires further clarification in line with recent efforts to clarify diagnostic criteria for malnutrition. This will improve consistency and advance research and improvements in nutrition systems and care to prevent HAM and improve patient health outcomes.

## Supplementary information

Appendix a. PRISMA 2020 Checklist

Appendix b. Search Strategy

## Data Availability

All data included in this systematic review was obtained from the studies included in Table 1. One author [33] provided the raw data to be able to extract the incidence of HAM. No other data collection was performed for this systematic review.

## References

[1] Alberda C, Graf A, McCargar L (2006). Malnutrition: etiology, consequences, and assessment of a patient at risk. Best Pr Res Clin Gastroenterol.

[2] Butterworth Jr Charles E (1974). The skeleton in the hospital closet. Nutr Today.

[3] Marshall S (2018). Why is the skeleton still in the hospital closet? a look at the complex aetiology of protein-energy malnutrition and its implications for the nutrition care team. J Nutr Health Aging.

[4] Cederholm T, Barazzoni R, Austin P, Ballmer P, Biolo G, Bischoff SC (2017). ESPEN guidelines on definitions and terminology of clinical nutrition. Clin Nutr.

[5] Cederholm T, Jensen GL, Correia MITD, Gonzalez MC, Fukushima R, Higashiguchi T (2019). GLIM criteria for the diagnosis of malnutrition – a consensus report from the global clinical nutrition community. J Cachexia Sarcopenia Muscle.

[6] White Jane V, Peggi G, Gordon J, Ainsley M, Marsha. S (2012). Consensus statement of the academy of nutrition and dietetics/American society for parenteral and enteral nutrition: characteristics recommended for the identification and documentation of adult malnutrition (undernutrition). J Acad Nutr Dietetics.

[7] Detsky Allan S, Baker JP, Johnston N, Whittaker S, Mendelson RA, Jeejeebhoy KN (1987). What is subjective global assessment of nutritional status?. J Parenter Enter Nutr.

[8] Ottery Faith D (1996). Definition of standardized nutritional assessment and interventional pathways in oncology. Nutrition.

[9] Vellas B, Guigoz Y, Garry PJ, Nourhashemi F, Bennahum D, Lauque S (1999). The Mini nutritional assessment (MNA) and its use in grading the nutritional state of elderly patients. Nutrition..

[10] Cheryl W, Allison F, Merrilyn B, Elisabeth I, Michelle M, Caitlin S (2009). Evidence based practice guidelines for the nutritional management of malnutrition in adult patients across the continuum of care. Nutr dietetics.

[11] Australian commision on Safety and Quality in Health Care. Hospital-Acquired Complications 13: Malnutrition2018. Available from: https://www.safetyandquality.gov.au.

[12] Watterson C, Fraser A, Banks M, Isenring E, Silvester C, Hoevenaars R, et al. Evidence based practice guidelines for the nutritional management of malnutrition in adult patients across the continuum of care. Nutr Dietetics. 2009;66:1–34.

[13] Agarwal E, Ferguson M, Banks M, Bauer J, Capra S, Isenring E. Nutritional status and dietary intake of acute care patients: results from The Nutrition Care Day Survey 2010. Clin Nutr. 2012;31:41–7. 10.1016/j.clnu.2011.08.002 21862187

[14] Merrilyn B, Susan A, Judy B, Deanne G (2007). Prevalence of malnutrition in adults in Queensland public hospitals and residential aged care facilities. Nutr Dietetics.

[15] Barker Lisa A, Gout Belinda S, Crowe Timothy C (2011). Hospital malnutrition: prevalence, identification and Impact on patients and the healthcare system. Int J Environ Res Public Health.

[16] Kahokehr Arman A, Tarik S, Kit W, Vahe S, Plank Lindsay D, Hill Andrew G (2010). Prevalence of malnutrition on admission to hospital–acute and elective general surgical patients. e-SPEN, Eur e-J Clin Nutr Metab.

[17] Correia M, Isabel TD, Campos Antonio Carlos L, Study ELAN Cooperative. (2003). Prevalence of hospital malnutrition in Latin America:: the multicenter ELAN study. Nutrition..

[18] McWhirter JP, Pennington CR (1994). Incidence and recognition of malnutrition in hospital. Bmj.

[19] Planas M, Audivert S, Pérez-Portabella C, Burgos R, Puiggrós C, Casanelles JM (2004). Nutritional status among adult patients admitted to an university-affiliated hospital in Spain at the time of genoma. Clin Nutr.

[20] Norman K, Pichard C, Lochs H, Pirlich M (2008). Prognostic impact of disease-related malnutrition. Clin Nutr.

[21] Cheng J, Witney-Cochrane K, Cunich M, Ferrie S, Carey S (2019). Defining and quantifying preventable and non-preventable hospital-acquired malnutrition-a cohort study. Nutr Diet.

[22] Correia MI, Hegazi RA, Diaz-Pizarro Graf JI, Gomez-Morales G, Fuentes Gutiérrez C, Goldin MF (2016). Addressing disease-related malnutrition in healthcare: a Latin American perspective. JPEN J Parenter Enter Nutr.

[23] Australian Commision on safety and quality in health care. the state of patient safety and quality in Asutralian hopsitals 2019. Available from: https://www.safetyandquality.gov.au/publications-and-resources/resource-library/state-patient-safety-and-quality-australian-hospitals-2019.

[24] Page Matthew J, McKenzie Joanne E, Bossuyt Patrick M, Isabelle B, Hoffmann Tammy C, Mulrow Cynthia D (2021). The PRISMA 2020 statement: an updated guideline for reporting systematic reviews. BMJ..

[25] Cederholm T, Jensen GL, Correia MITD, Gonzalez MC, Fukushima R, Higashiguchi T (2019). GLIM criteria for the diagnosis of malnutrition – A consensus report from the global clinical nutrition community. Clin Nutr.

[26] National centre for classification in health. The international statistical classification of diseases and related health problems, tenth revision, Australian modification (ICD-10-AM). 10th revision, Australian modification, 8th ed. Wollongong, NSW: National Casemix & Classification Centre, University of Wollongong; 2013.

[27] Academy of nutrition and dietetics. Evidence analysis manual: steps in the Academy Evidence Analysis Process. 2016. Available from: https://www.andeal.org/evidence-analysis-manual.

[28] Hung YC, Bauer J, Horsley P, Waterhouse M, Bashford J, Isenring E (2013). Changes in nutritional status, body composition, quality of life, and physical activity levels of cancer patients undergoing autologous peripheral blood stem cell transplantation. Support Care Cancer.

[29] Talia W, Christine J, Lynda R, Jan H, Breanne H, Fiona N (2020). A retrospective study of the incidence and characteristics of long-stay adult inpatients with hospital-acquired malnutrition across five Australian public hospitals. Eur J Clin Nutr.

[30] Gordon G, Oxman Andrew D, Akl Elie A, Kunz R, Vist G, Brozek J (2011). GRADE guidelines: 1. Introduction—GRADE evidence profiles and summary of findings tables. J Clin Epidemiol.

[31] DerSimonian R, Nan L (1986). Meta-analysis in clinical trials. Controlled Clin Trials.

[32] Higgins Julian PT, Thompson Simon G, Deeks Jonathan J, Altman Douglas G (2003). Measuring inconsistency in meta-analyses. BMJ.

[33] Cansado P, Ravasco P, Camilo M (2009). A longitudinal study of hospital undernutrition in the elderly: comparison of four validated methods. J Nutr Health Aging.

[34] Collins J, Porter J, Truby H, Huggins CE (2016). How does nutritional state change during a subacute admission? findings and implications for practice. Eur J Clin Nutr.

[35] Hejazi N, Mazloom Z, Farid Z, Abbas R, Afshin A (2016). Nutritional assessment in critically ill patients. Iran J Med Sci.

[36] Lin T, Yang J, Hong X, Yang ZY, Ge T, Wang M (2020). Nutritional status in patients with advanced lung cancer undergoing chemotherapy: a prospective observational study. Nutr cancer.

[37] Nip WFR, Perry L, McLaren S, Mackenzie A (2011). Dietary intake, nutritional status and rehabilitation outcomes of stroke patients in hospital. J Hum Nutr dietetics.

[38] Jin SE, Sun LJ, Yeon KJ (2012). Nutritional intake and nutritional status by the type of hematopoietic stem cell transplantation. Clin Nutr Res.

[39] Allard JP, Keller H, Jeejeebhoy KN, Laporte M, Duerksen DR, Gramlich L (2016). Decline in nutritional status is associated with prolonged length of stay in hospitalized patients admitted for 7 days or more: a prospective cohort study. Clin Nutr.

[40] Bell J, Bauer J, Capra S, Pulle Chrys R (2013). Barriers to nutritional intake in patients with acute hip fracture: time to treat malnutrition as a disease and food as a medicine?. Can J Physiol Pharm.

[41] Braunschweig C, Gomez S, Sheean PM (2000). Impact of declines in nutritional status on outcomes in adult patients hospitalized for more than 7 days. J Am Diet Assoc.

[42] Lima J, Teixeira PP, Eckert IDC, Burgel CF, Silva FM (2021). Decline of nutritional status in the first week of hospitalisation predicts longer length of stay and hospital readmission during 6-month follow-up. Br J Nutr.

[43] van Vliet Iris MY, Gomes-Neto António W, de Jong Margriet FC, Jager-Wittenaar H, Navis Gerjan J (2020). High prevalence of malnutrition both on hospital admission and predischarge. Nutrition..

[44] McDougall Karen E, Cooper PL, Stewart AJ, Huggins CE (2015). Can the mini nutritional assessment (MNA®) be used as a nutrition evaluation tool for subacute inpatients over an average length of stay?. J Nutr Health Aging.

[45] Whitley A, Skliros E, Graven C, McIntosh R, Lasry C, Newsome C (2017). Changes in nutritional and functional status in longer stay patients admitted to a geriatric evaluation and management unit. J Nutr Health Aging.

[46] Palmer M, Hill J, Hosking B, Naumann F, Stoney R, Ross L, et al. Quality of nutritional care provided to patients who develop hospital acquired malnutrition: a study across five Australian public hospitals. J Hum Nutr Diet. 2021;4:695–704. 10.1111/jhn.12876 33855787

[47] Collins JC. Malnutrition in subacute care. Thesis. Monash University. Melbourne, Australia. 2017.

[48] Marshall S, Bauer J, Isenring E (2014). The consequences of malnutrition following discharge from rehabilitation to the community: a systematic review of current evidence in older adults. J Hum Nutr Diet.

[49] Agarwal E, Ferguson M, Banks M, Batterham M, Bauer J, Capra S (2013). Malnutrition and poor food intake are associated with prolonged hospital stay, frequent readmissions, and greater in-hospital mortality: results from the Nutrition Care Day Survey 2010. Clin Nutr.

[50] Banks Merrilyn D, Nicholas G, Bauer Judith D, Ash S (2010). The costs arising from pressure ulcers attributable to malnutrition. Clin Nutr.

[51] Correia MI, Waitzberg DL (2003). The impact of malnutrition on morbidity, mortality, length of hospital stay and costs evaluated through a multivariate model analysis. Clin Nutr.

[52] Lin LS, Benjamin OKC, Huak CY, Chiong LW, Maree F, Lynne D (2012). Malnutrition and its impact on cost of hospitalization, length of stay, readmission and 3-year mortality. Clin Nutr.

[53] Independent Hospital Pricing Authority. Pricing and funding for safety and quality. Risk adjustment model for hospital acquired complications. Version 3. 2018 Available from: https://www.ihpa.gov.au/.

[54] Independent Hospital Pricing Authority. Consultation paper on the pricing framework for australian public hospital services 2019–2020. 2019. https://www.ihpa.gov.au/.

[55] Jensen Gordon L, Bruce B, Ronenn R, Heimburger Douglas C (2009). Malnutrition syndromes: a conundrum vs continuum. JPEN J Parenter Enter Nutr.

[56] Cederholm T, Bosaeus I, Barazzoni R, Bauer J, Van Gossum A, Klek S (2015). Diagnostic criteria for malnutrition – an ESPEN consensus statement. Clin Nutr.

