# Supplementary material for: Incidence and criteria used in the diagnosis of hospital-acquired malnutrition in adults: a systematic review and pooled incidence analysis
Source: Eur J Clin Nutr. 2022 May 2;77(1):23–35. doi: 10.1038/s41430-022-01141-2 (PMC9876784; doi:10.1038/s41430-022-01141-2)
Supplement: Supplementary file 2 — Appendix b. Search Strategy [file 41430_2022_1141_MOESM2_ESM.docx]

**Appendix b. Search strategy for: Incidence and Criteria used in the Diagnosis of Hospital-Acquired Malnutrition in Adults: A Systematic Review and Pooled Incidence Analysis**

The literature search was conducted several times until all the databases produced the most relevant results. All of the search strategies were assisted by the Queensland University’s Librarian located at Herston (Royal Brisbane and Women’s Hospital). The chosen search strategy is as shown below.

**PubMed, results: 1574**

((("hospital-acquired malnutrition"[tiab] OR "hospital acquired malnutrition"[tiab] OR "hospital malnutrition"[tiab] OR "nosocomial malnutrition" [tiab]) OR ("nutritional status"[tiab] AND ("decline"[tiab] OR "change"[tiab] OR "changes"[tiab] OR "deterioration"[tiab]) AND ("Hospitalization"[Mesh] OR "hospitalization"[tiab] OR "hospitalisation"[tiab] OR "hospital"[tiab] OR "Hospitals"[Mesh] OR "hospitalised"[tiab] OR "hospitalized"[tiab] OR "nosocomial"[tiab] OR "Inpatients"[Mesh] OR "inpatients"[tiab] OR "in-patients"[tiab]))) NOT (("child" OR "childs" OR "children" OR "pediatrics"))) NOT (("animal" OR "animals"))

**CINAHL (via EBSCOhost), results: 493**

TI ( ("hospital-acquired malnutrition" OR "hospital acquired malnutrition" OR "hospital malnutrition" OR "nosocomial malnutrition") ) OR AB ( ("hospital-acquired malnutrition" OR "hospital acquired malnutrition" OR "hospital malnutrition" OR "nosocomial malnutrition") ) OR TI ( ("nutritional status" AND ("decline" OR "change" OR "changes" OR "deterioration")) ) OR AB ( ("nutritional status" AND ("decline" OR "change" OR "changes" OR "deterioration")) )) AND (TI ( "hospitalization" OR "hospitalisation" OR "hospital" OR "hospitalised" OR "hospitalized" OR "nosocomial" OR "Inpatients"[Mesh] OR "inpatients" OR "in-patients" ) OR AB ( "hospitalization" OR "hospitalisation" OR "hospital" OR "hospitalised" OR "hospitalized" OR "nosocomial" OR "Inpatients"[Mesh] OR "inpatients" OR "in-patients" ) OR (MH "Hospitals+") OR (MH "Hospitalization+") OR (MH "Inpatients")) NOT TX ("child" OR "childs" OR "children" OR "pediatrics") NOT TX ("animal" OR "animals")

Limiters - Published Date: 19900101-20201231
Expanders - Apply equivalent subjects
Narrow by Subject Age: - all adult
Search modes - Boolean/Phrase

**Embase, results: 1643**

Search Strategy:

1. 'child' OR 'childs' OR 'children' OR 'pediatrics'
2. 'animal' OR 'animals'
3. ('hospital-acquired malnutrition':ti,ab OR 'hospital acquired malnutrition':ti,ab OR 'hospital malnutrition':ti,ab OR 'nosocomial malnutrition':ti,ab OR ('nutritional status':ti,ab AND ('decline':ti,ab OR 'change':ti,ab OR 'changes':ti,ab OR 'deterioration':ti,ab))) AND ('hospital'/exp OR 'hospitalization'/exp OR 'hospital patient'/exp OR 'hospitalization':ti,ab OR 'hospitalisation':ti,ab OR 'hospital':ti,ab OR 'hospitalised':ti,ab OR 'hospitalized':ti,ab OR 'nosocomial':ti,ab OR 'inpatients':ti,ab OR 'in-patients':ti,ab)
4. ('hospital-acquired malnutrition':ti,ab OR 'hospital acquired malnutrition':ti,ab OR 'hospital malnutrition':ti,ab OR 'nosocomial malnutrition':ti,ab OR ('nutritional status':ti,ab AND ('decline':ti,ab OR 'change':ti,ab OR 'changes':ti,ab OR 'deterioration':ti,ab))) AND ('hospital'/exp OR 'hospitalization'/exp OR 'hospital patient'/exp OR 'hospitalization':ti,ab OR 'hospitalisation':ti,ab OR 'hospital':ti,ab OR 'hospitalised':ti,ab OR 'hospitalized':ti,ab OR 'nosocomial':ti,ab OR 'inpatients':ti,ab OR 'in-patients':ti,ab) (***repeated by mistake in the search engine)***
5. #3 NOT #1 NOT #2
6. #5 AND ('article'/it OR 'article in press'/it OR 'review'/it)
7. Search 6 was further limited by publication type by excluding conference abstracts

**
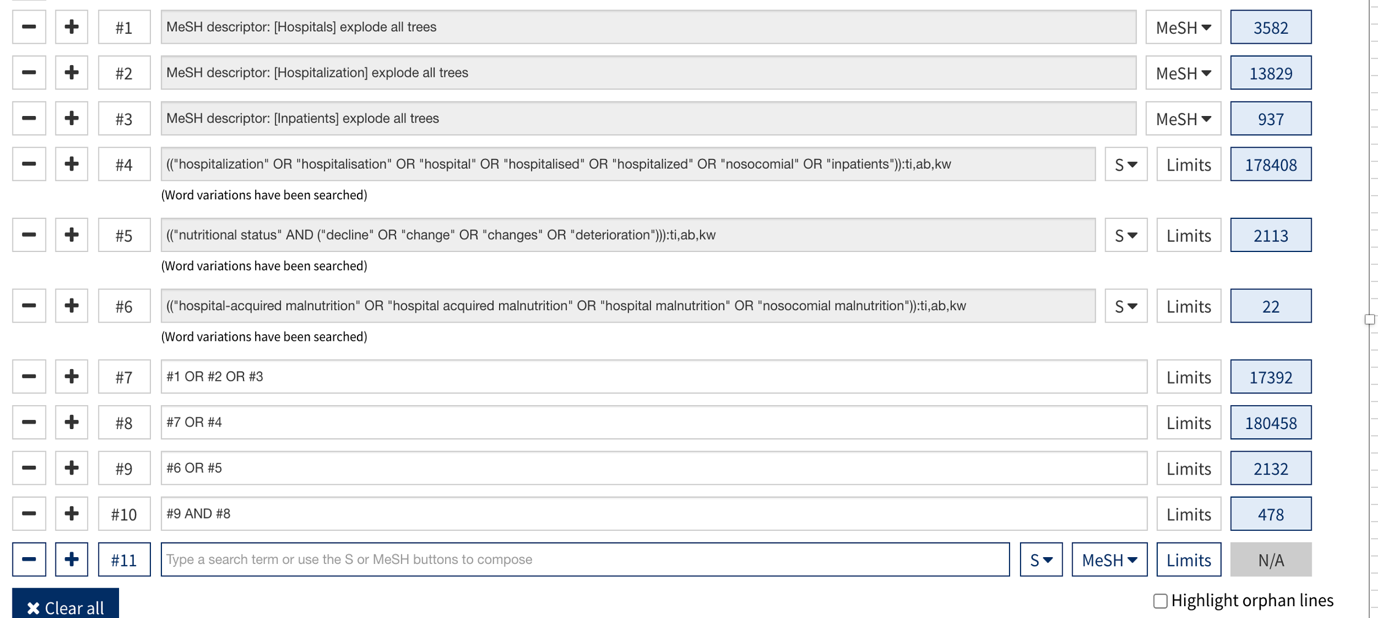
Cochrane, results: 466**
